# Prevalence and risk factors for infection of bovine tuberculosis in indigenous cattle in the Serengeti ecosystem, Tanzania

**DOI:** 10.1186/1746-6148-9-267

**Published:** 2013-12-30

**Authors:** Bugwesa Z Katale, Erasto V Mbugi, Esron D Karimuribo, Julius D Keyyu, Sharon Kendall, Gibson S Kibiki, Peter Godfrey-Faussett, Anita L Michel, Rudovick R Kazwala, Paul van Helden, Mecky I Matee

**Affiliations:** 1 Department of Microbiology and Immunology, School of Medicine, Muhimbili University of Health and Allied Sciences (MUHAS), P.O BOX 65001, Dar es Salaam, Tanzania; 2 Tanzania Wildlife Research Institute (TAWIRI), P.O BOX 661, Arusha, Tanzania; 3 Department of Veterinary Medicine and Public Health, Faculty of Veterinary Medicine, Sokoine University of Agriculture (SUA), P.O BOX 3021, Morogoro, Tanzania; 4 Centre for Emerging, Endemic and Exotic diseases, Royal Veterinary College (RVC), Hawkshead Lane, North Mymms, Hatfield, Hertfordshire AL9 7TA, UK; 5 Kilimanjaro Christian Medical College, Kilimanjaro Clinical Research Institute (KCRI), Tumaini University, P.O. BOX 2240, Moshi, Tanzania; 6 Department of Infectious and Tropical Diseases, London School of Hygiene and Tropical Medicine (LSHTM), London, UK; 7 Department of Veterinary Tropical Diseases, Faculty of Veterinary Sciences, University of Pretoria, Private Bag X4, Onderstepoort 0110, South Africa; 8 DST/NRF Centre of Excellence for Biomedical Tuberculosis Research/MRC Centre of Molecular and Cellular Biology, Division of Molecular Biology and Human Genetics, Faculty of Health Sciences, University of Stellenbosch, Tygerberg, Capetown, South Africa

**Keywords:** Risk factors, Bovine tuberculosis, *Mycobacterium bovis*, Human-animal interface, Serengeti ecosystem, Wildlife

## Abstract

**Background:**

Bovine tuberculosis (bTB) is a chronic debilitating disease and is a cause of morbidity and mortality in livestock, wildlife and humans. This study estimated the prevalence and risk factors associated with bovine tuberculosis transmission in indigenous cattle at the human-animal interface in the Serengeti ecosystem of Tanzania.

**Results:**

A total of 1,103 indigenous cattle from 32 herds were investigated for the presence of bTB using the Single Intradermal Comparative Tuberculin Test. Epidemiological data on herd structure, management and grazing system were also collected.

The apparent individual animal prevalence of tuberculin reactors was 2.4% (95% confidence interval (CI), 1.7 – 3.5%), whereas the true prevalence was 0.6% CI, 0.6 – 0.7% as indicated by a reaction to avian tuberculin purified protein derivatives (PPD) which is more than 4 mm greater than the reaction to avian tuberculin PPD. The results showed that 10.6% (117/1,103) showed non-specific reactions (atypical mycobacterium). The herd prevalence of 50% (16/32) was found. Tuberculin skin test results were found to be significantly associated with age, location, size of the household and animal tested. Of 108 respondents, 70 (64.8%) individuals had not heard about bovine tuberculosis at all. Thirty five percent (38/108) of respondents at least were aware of bTB. About 60% (23/38) of respondents who were aware of bTB had some knowledge on how bTB is spread. Eighty one percent (87/108) of respondents were not aware of the presence of bTB in wildlife. There is regular contact between cattle and wild animals due to sharing of grazing land and water sources, with 99% (107/108) of households grazing cattle in communal pastures.

**Conclusion:**

The study has demonstrated a high reported interaction of livestock with wildlife and poor knowledge of most cattle owners concerning bTB and its transmission pathways among people, livestock and wildlife. Although the overall proportion of animals with bTB is relatively low, herd prevalence is 50% and prevalence within herds varied considerably. Thus there is a possibility of cross transmission of bTB at wildlife-livestock interface areas that necessitates use of genetic strain typing methods to characterize them accurately.

## Background

Bovine tuberculosis (bTB) caused by *Mycobacterium bovis,* is a chronic debilitating disease of livestock, wildlife and humans [1,2]. Cattle may serve as the main host for *M. bovis* worldwide [3], while many or most other species such as possums, pigs, cats, dogs, horses and sheep are considered to be spill-over hosts [4]. Aerosol is considered to be the main route of infection in animals [3,4]. Other routes of infection such as ingestion of contaminated feeds, water and fomites have been identified [3]. Information concerning routes of transmission and different potential sources of infection in Africa is scarce [5].

Bovine tuberculosis is a disease with potential public health and economic importance [6] since it can affect international trade of animals and animal products [7]. The presence of bTB in domesticated and wild animals in synergy with the HIV pandemic in developing countries makes zoonotic tuberculosis a potential threat to human health [1,8]. In developed countries, bTB has been controlled through ‘a test-and slaughter policy’. Nevertheless, bTB remains a problem in most developing countries where surveillance and control activities are often inadequate or unavailable [6] possibly due to lack of funds to support the whole exercise and compensate for tested and slaughtered animals in these countries.

Previously published information indicates that bTB is endemic in Tanzania’s cattle, with regional prevalences ranging from 0.2% to 13.2% [5,9-15] suggesting the presence of foci of infection [13]. This could be underestimated if not confirmed by currently available bacteriological or molecular techniques [16]. A high prevalence (13.2%) of bTB was reported in pastoral cattle in the southern highlands of Tanzania and was associated with high numbers of indigenous cattle kept under intensive husbandry practice [17]. In Tanzania, *M. bovis* has been isolated from human lymph biopsies [18,19], cow’s milk and tissue samples from slaughter houses [12,19] and from a range of wildlife species including migratory wildebeest, topi and lesser kudu [20]. In their study, Renwick et al. [21] revealed bTB to be established in domestic stock and recently native wild bovids particularly African buffalo has been infected. In Tanzania, up-to-date information concerning risk factors for transmission of bTB at the livestock-wildlife interface is lacking. Studies conducted in the Southern Highlands and northern parts of the country reported an association of bTB infection with age, sex, breed, lactation and variation in climate [17] and proximity to wildlife [5]. Likewise, studies conducted in other African countries and elsewhere reported an association of bTB infection with age, sex, physiological status and husbandry practices such as cattle movement and contact with wildlife [22-26].

The Serengeti ecosystem comprises an ecosystem defined by annual movement of herds of ungulates [27] interacting with populations of livestock. The husbandry practice of most of the pastoral communities in the region is pastoralism, based on *transhumance*, which refers to a pattern of seasonal movement between dry season and wet season pastures. Given the husbandry practices and proximity to wildlife, studies are needed to explore the disease status, dynamics in the ecosystem and risk factors for bTB infection in animals and ultimately also humans. Here we present findings of a study conducted to determine the prevalence of bTB and risk factors associated with bTB infection in pastoral and agro-pastoral communities at the livestock-wildlife interface in the Serengeti ecosystem in Tanzania.

## Methods

### Study site

**Figure 1:**
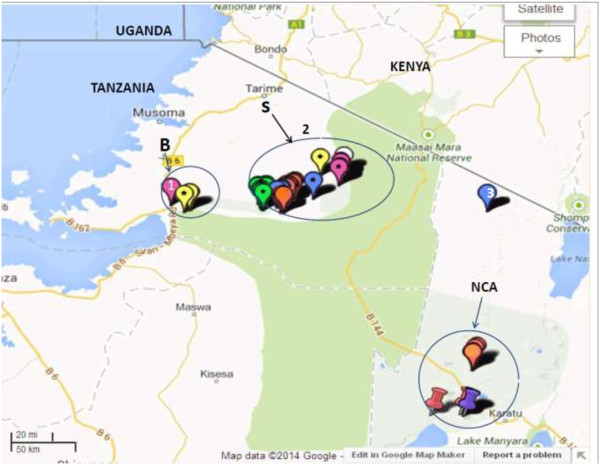
**Map of the Serengeti ecosystem and its surrounds, showing study sites in Bunda, Serengeti and Ngorongoro districts, Tanzania.** 1: Bunda district headquarter (HQ); 2: Serengeti district HQ; 3: Ngorongoro district HQ; B, S and NCA are coordinates of study sites where data on Single Intradermal Comparative Tuberculin Test and questionnaires were obtained in Bunda (B), Serengeti (S) and Ngorongoro Conservation Area (NCA). Source: Map data *@*2014, Google.

The study was carried out in the Serengeti and Bunda districts of the Mara region, and the Ngorongoro district of Arusha region in northern Tanzania, from October to December 2011 (Figure 1). Serengeti (S2° 0' 0, E 34° 49' 60) and Bunda districts (S 2° 0' 0, E33° 49' 60) are two out of five districts in the Mara region, which are part of the western Serengeti with a rapidly growing human population, and concomitant land pressure. The human population in Bunda and Serengeti districts is estimated to be 260,000 (91.3 persons per km^2^) and 176,609 (16.1 persons per km^2^), respectively [28]. The western Serengeti receives an annual rainfall between 500–1200 mm, declining eastwards towards the park boundary and increasing towards Lake Victoria [29,30]. The western Serengeti receives two rainy seasons; the long rains occurring between March to May, and the short rains from October to November. These two districts border the Serengeti ecosystem on the western side and are dominated by smallholder agro-pastoralist communities whose activities depend on agriculture and livestock as a source of income. The agro-pastoral communities in Bunda and Serengeti districts have permanent settlements and keep local cattle (Zebu) managed in an extensive grazing system.

The Ngorongoro Conservation Area (NCA) (8,288 km^2^) in the Ngorongoro district (E35° and 36°E and S 2° and 4°), of the Arusha region is part of the Serengeti ecosystem extending from the plains of Serengeti National Park (SNP) in the north-west, to the eastern arm of the Great Rift Valley. Both SNP and NCA are part of the UNESCO (United Nations Educational, Scientific and Cultural Organization) Biosphere Reserve. The Ngorongoro district has a population of 129,776 (9.6 persons per km^2^) [28]. NCA has a high local diversity of climate resulting from extensive variation in relief and the dynamics of air masses. The variation in climate has resulted in distinct habitats, comprising dense montane forest cover on the steep slopes of the crater, open grass plains with alternating fresh and brackish water lakes, swamps and two patches of acacia woodland; Lerai Forest comprising dominant tree species, *Acacia xanthonhloea* and *Rauvolfia caffra.* The conservation area is dominated by pastoralists of the Maasai ethnic group who constantly move livestock in search of pasture and water where interaction with wildlife is common. Infection of cattle with bTB poses a great risk to infection in wildlife in Ngorongoro due to great interaction between cattle and wildlife as the Maasai pastoralists are found within the conservation area [31]. Rainfall is seasonal in Ngorongoro district and follows the altitudinal gradient. Annual precipitation on the arid plains varies from 500 mm in the west to 1700 mm along the forested slopes in the east.

### Sample size calculation

The sample size for the study was calculated by using StatsDirect statistical software version 2.7.8 at 95% confidence interval based on 13.2% as the highest prevalence of bTB in indigenous cattle in Tanzania [12] with 2% as an acceptable absolute deviation of sample rate from population rate. The population of cattle in three districts was estimated to be 1,045,000 (personal communication, District Veterinary Officers). Based on the above calculation, the total number of animals estimated for testing was 1100.

### Study design and subjects

The study was a cross sectional study conducted in villages bordering the Serengeti ecosystem in Tanzania. A complete list by names of all villages bordering the Serengeti ecosystem in Bunda and Serengeti districts was obtained from the District Agriculture and Livestock authorities. Likewise, a list of all villages inside the Ngorongoro Conservation Area was obtained from Ngorongoro Conservation Area Authority (NCAA) veterinary unit. A convenience sample of 11 villages was selected based on their proximity to wildlife, the willingness of the pastoral community to participate in the study and availability of Veterinary Services staff experienced to perform TB testing of cattle. In some areas, experience of the local veterinary service staff with the community and nomadic lifestyle of the pastoral communities dictated the selection of herds for inclusion. A total number of 32 herds were thus available for tuberculin skin testing. For herds with cattle ranging between 1–50 herds, every 2^nd^ animal was tested for bTB, every 4^th^ animal was tested in the case of herds of 51–200 animals and every 12^th^ animal was tested for bTB in cattle enclosures with herd size ≥201. This was done by gathering the cattle into an enclosure (boma or kraal) and allowing the cattle to exit one at a time. Every 2^nd^, 4^th^ or 12^th^ animal was then selected for study. The procedure resulted into 292, 289 and 522 cattle in each group (1–50, 51–200 and ≥201, respectively), which were finally tested for bTB leading to a total number of 1111 cattle. Readings from eight animals (8) animals could not be obtained for post PPD injection reading as the animals were not available. Regardless of gender all cattle older than 6 months were restrained by using ropes with different restraint techniques depending on the prevailing situation. Body condition in individual animals was assessed using a modified guideline described by Msangi et.al. [32], where animals were classified as emaciated (score 1), thin (score 2), normal (score 3), musculous (score 4), and fat (score 5).

### Single intradermal comparative tuberculin test

Tuberculin skin testing was performed using aliquots of 0.1 mL of 2500 IU/mL bovine purified protein derivative (PPD) and 0.1 mL of 2500 IU/mL avian PPD (Prionics Lelystad B.V, Lelystad , The Netherland). Bovine and avian PPDs were injected intradermally at two sites approximately 12 centimetres apart at the border of the anterior and middle thirds of one side of the neck. This was done after shaving the two sites by using a razor blade. The skin thickness was measured with callipers prior to and 72 hours after PPD injection and recorded. For young animals where there is insufficient space to inject both tuberculin PPDs into the same side of the neck, the tuberculin PPDs were injected on different sides of the neck. Clinically sick animals and cows one month pre-and post-partum were excluded from the skin test considering the expected clearly weaker cellular immune response that might result into false negative for the tuberculin test in this group of animals. Bovine positive reactors and avian reactors were identified using the following formulae (BOV_72_ – BOV_0_) – (AV_72_ – AV_0_) and (AV_72_ – AV_0_) - (BOV_72_ – BOV_0_) respectively, where BOV_0_ and AV_0_ and BOV_72_ and AV_72_ indicate skin thicknesses prior and 72 hrs post injection of bovine and avian tuberculin respectively [13]. Interpretation of skin reactions of the SCITT test was based on recommendation by the manufacturers (Prionics Lelystad B.V, Lelystad, The Netherland). Positive: A reaction to bovine tuberculin PPD, avian tuberculin which is more than 4 mm greater than the reaction to avian tuberculin PPD, and presence of clinical signs (In our case, due to disease endemicity, a reading more than 4 mm greater than the reaction to avian tuberculin PPD was considered positive regardless of presence or absence of clinical signs). Inconclusive: A reaction to bovine tuberculin PPD (of at least 2 mm) which is from 1–4 mm greater than the reaction to avian tuberculin PPD, and absence of clinical signs. Negative: A reaction to bovine tuberculin PPD which is equal or less than the reaction to avian tuberculin PPD, and the absence of clinical signs.

### Household questionnaire survey

**Figure 2:**
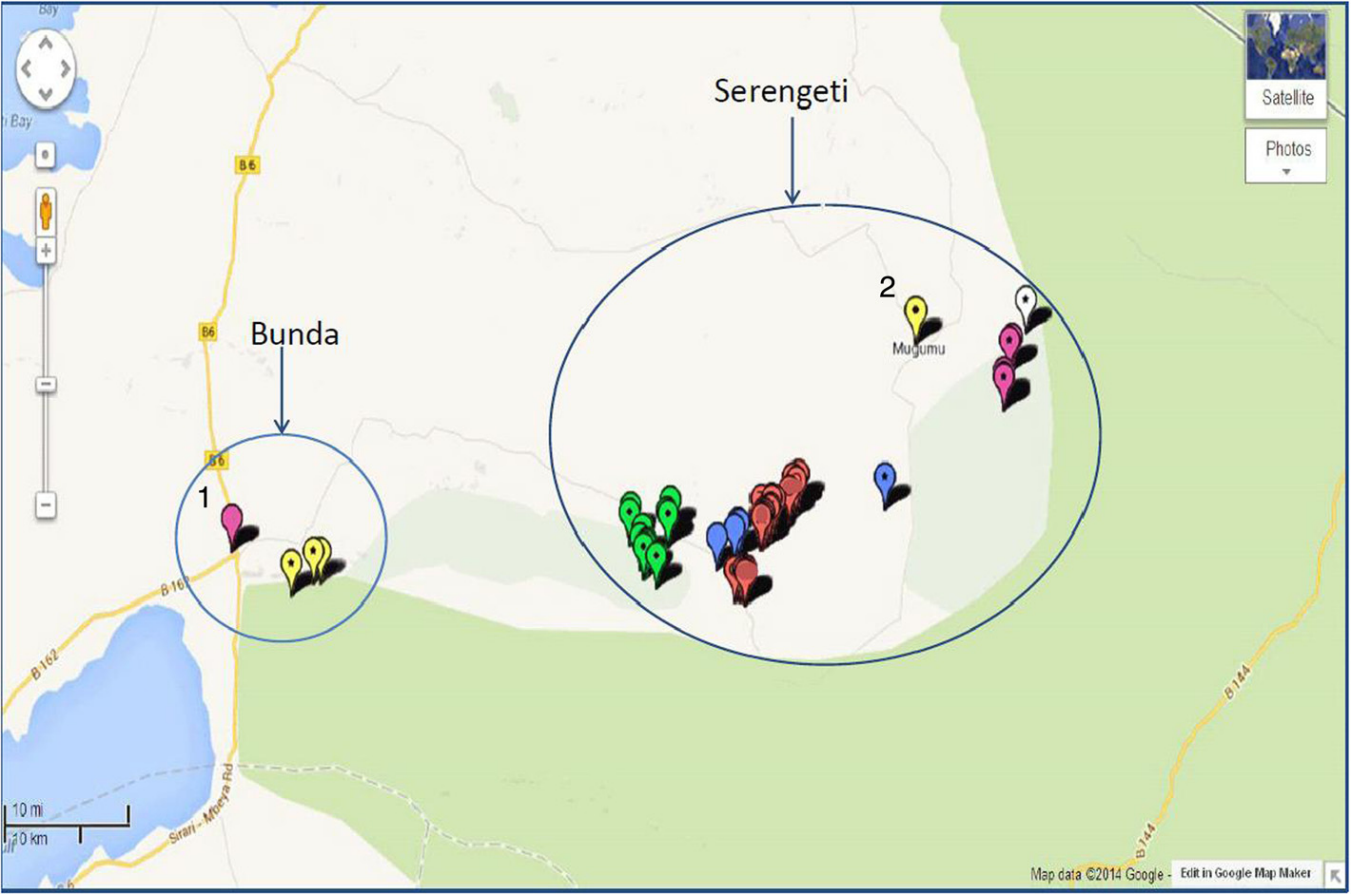
**Map of the Bunda and Serengeti districts showing coordinates in villages where tuberculin and questionnaire survey was conducted 1: Bunda district headquarter (HQ); 2: Serengeti district headquarter (HQ).** Source: Map data *@*2014, Google.

**Figure 3:**
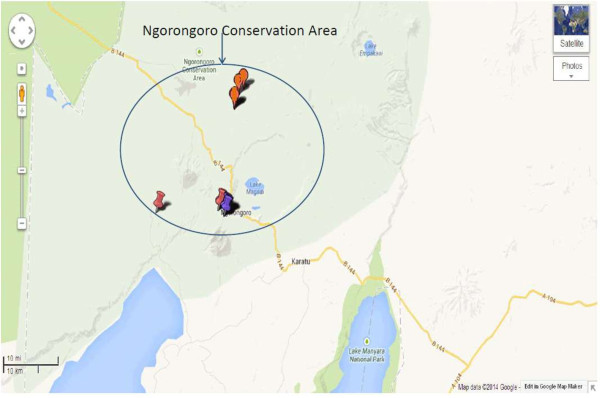
**Map of the Ngorongoro conservation area showing coordinates where single intradermal comparative tuberculin test and questionnaire survey was conducted.** Source: Map data *@*2014, Google.

The questionnaire survey was conducted to assess awareness of cattle owners and to identify the role of potential risk factors for bovine tuberculosis infection among cattle. The willingness and cooperation of the pastoral community to participate in the questionnaire survey was used to select number of households. A total of 108 cattle owners comprised of 32 households where tuberculin skin testing was conducted and a convenience sample of 76 additional households that kept cattle but were not available for us to perform tuberculin skin testing were interviewed but thought that their information was useful and valuable to have an overview of awareness and role of risk factors on transmission of bTB. A face to face interview in Kiswahili language was administered by using a Smartphone [33], In circumstances where Kiswahili language was not a language of communication, a translator familiar with the local language was used. The interview was conducted concerning tuberculin skin testing in cattle. A pre-tested “close-ended” questionnaire form comprising of variables such as family size, breakdown of herd size and structure, herd management including veterinary services, cattle movement, cattle feeding patterns, wildlife contact and movements and knowledge on bovine tuberculosis as described by Munyeme et al. [34] was used. In addition, data on owner’s family size and data on individual animal such as sex and age were recorded. Animal data including pictures of representative cows indicating their health status; and location (GPS) (Figures 2 and 3) was recorded via EpiCollect from a smartphone and saved to the central database server located at Southern African Centre for Infectious Diseases Surveillance (SACIDS), Sokoine University of Agriculture (SUA) in Morogoro, Tanzania.

### Ethical consideration

This study was approved by the National Institute of Medical Research (NIMR), Tanzania (Reference number NIMR/HQ/R.8a/Vol.IX/1299).

### Data analysis

The data from the questionnaire were entered and analysed using SPSS for Windows (version 17.0. SPSS, Inc., Chicago, IL, USA). The apparent prevalence of bovine tuberculosis was defined as the number of positive reactors divided by the number of cattle in which the test was read. True prevalence was calculated based on formula described by Rogan and Gladen [35], TP = (AP + SP-1)/(SE + SP-1); where TP is true prevalence, AP the apparent prevalence, SE is sensitivity, and SP is specificity. Metaanalysis of sensitivity and specificity results that were used to calculate true prevalence were pooled from Amen et al. [36], Muller et al. [37] and Quirin et al. [38]. Based on these studies a sensitivity and specificity of 59% and 95% respectively were established and therefore used to calculate the true prevalence in this study. The confidence interval for true and apparent prevalence was calculated at 95% using confidence interval for population proportion (Confidence Interval Calculator [version 4, November, 2002]; http://vl.academicdirect.org/applied_statistics/binomial_distribution/ref/CIcalculator.xls). Herd level prevalence was calculated as the number of herds with at least one-reactor positive animal divided by the total number of herds tested. General linear models was used to assess the association between the different risk factors (age, sex, herd size, location, size of household and animal tested) and results of single intradermal comparative tuberculin test (SICTT) of individual animals using STATA for Windows (version 12.0; StataCorp, 4905 Lakeway Drive College Station, Texas 77845 USA, 800-STATA-PC) with a *p*-value of <0.05 considered statistically significant. In both univariate and multivariate analysis, a random effect logistic regression analysis was performed with herd treated as a random effect to account for the difference in herd sizes and also for the fact that animals within herd could be considered as forming a cluster. In univariate analysis variables with *p*-value ≤ 0.25 [39] and those of known plausible biological contribution for bTB positivity were carried out for multivariate analysis. In multivariate analysis, a forward selection approach was then used to include variables from the model based on a likelihood ratio test. Descriptive statistics were used to test whether herds were normally distributed or not and non parametric test (Kruskal-Wallis Test) was used for prediction of prevalence by herds.

## Results

A total number of 1111 cattle were screened for bovine tuberculosis. Eight (8) cattle were not found during follow up for the second reading after 72 hours. The apparent individual animal prevalence of tuberculin reactors was 2.4% (95% confidence interval (CI), 1.7 – 3.5%, whereas the true prevalence was 0.6% (95% CI), 0.6 – 0.7%. The apparent prevalence was comprised of 3.17% (12 out of 379) males and 2.07% (15 out of 724) females (Table 1). A herd prevalence of 50% (16 out of 32) was recorded and prevalence of individual herds varies and ranges from 0 to 9.5%. The prevalence of non-specific infection (atypical mycobacteria) was 10.6% (117 out of 1103).

**Table 1:** Univariate analysis of risk factors for cattle tuberculin reactors using General Linear models (GLM) with herd as random effect

**Risk factors**	**Proportional% (No/total)**	**OR**	**95% CI**	**( *p *-value)**
Sex				
Female	2.07 (15/724)	1.00*	-	-
Male	3.17 (12/379)	1.53	0.714; 3.29	*0.27*
Age				
<2 years	1.30 (3/231)	1.00*	-	-
2–4 years	3.6 (11/304)	11.10	1.449; 85.062	*0.02*
Over 4 years	2.3 (13/568)	4.96	0.642; 38.398	0.67
Location				
Serengeti	2.64 (15/569)	1.00*	-	-
Ngorongoro	2.94 (11/374)	1.12	0.508; 2.464	*0.78*
Bunda	0.63 (1/160)	0.23	0.030; 1.772	*0.16*
Animal tested				
1–20	3.5 (6/170)	1.00*	-	***-***
21–40	1.9 (5/265)	0.76	0.229; 2.545	*0.66*
≥ 41	2.4 (16/668)	0.81	0.292; 2.243	*0.69*
Household size				
1–5	1.30 (2/154)	1.00*	-	-
6–10	2.4 (6/248)	1.12	0.369;3.413	*0.84*
> = 11	2.7 (19/701)	0.56	0.198;1.603	*0.28*

OR: Odds ration; CI: Confidence interval; *p*: p-value; *Reference level.

In multivariate analysis all variables resulting from univariate analysis were carried on for further analysis. Results from multivariate analysis indicate that, the risk factors (Age, location, herd size, animal tested and size of the household; Table 2) which were considered into this study significantly contributed to positive reactivity to bTB. As indicated in Table 2, sex of animals was not significantly associated with the result of Single Intradermal Comparative Tuberculin Test of individual cattle (OR = 1.53; CI; 0.71 – 3.29). This reflects that males were 1.5 times more likely to test positive than females. As regards to significant association depicted in result of intradermal skin positivity with location, the highest prevalence of bTB was found in Ngorongoro district (2.94%), followed by Serengeti (2.64%) and Bunda district (0.63%).

**Table 2:** Multivariate analysis of risk factors for cattle tuberculin reactors using General Linear models (GLM) with herd as random effect

**Risk factors**	**Proportional% (No/total)**	**OR**	**95% CI**	**( *p *-value)**
Sex				
Female	2.07 (15/724)	1.00*	-	-
Male	3.17 (12/379)	1.53	0.714; 3.29	*0.274*
Age				
<2 years	1.30 (3/231)	1.00*	-	-
2–4 years	3.6 (11/304)	11.06	1.444; 84.785	0.005
Over 4 years	2.3 (13/568)	5.71	0.717; 45.436	
Location				
Serengeti	2.64 (15/569)	1.00*	-	-
Ngorongoro	2.94 (11/374)	0.93	0.417; 2.077	0.007
Bunda	0.63 (1/160)	0.21	0.028; 1.634	
Animal tested				
1–20	3.5 (6/170)	1.00*	-	***-***
21–40	1.9 (5/265)	0.55	0.151; 2.006	
≥ 41	2.4 (16/668)	0.88	0.308; 2.534	0.015
Household size				
1–5	1.30 (2/154)	1.00*	-	-
6–10	2.4 (6/248)	1.97	0.599; 6.478	
> =11	2.7 (19/701)	0.74	0.255; 2.163	0.007

OR: Odds ration; CI: Confidence interval; *p*: p-value (Likelihood ratio test); *Reference level.

**Figure 4:**
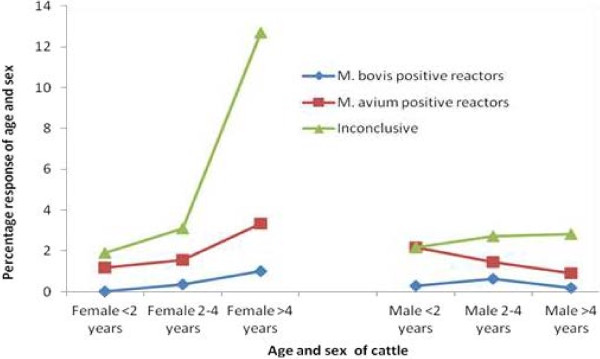
The variation of skin reaction to single intradermal comparative tuberculin test with age and sex.

The variation of tuberculin skin reaction in relation to age and sex is shown in Figure 4. The positivity to doubtful results increased with age in both sexes, while positivity to *M. bovis* seems to increase with age in females but not in males. Female cattle over 4 years (>4 years) had a high prevalence of *M. bovis* compared to other groups. The questionnaire results (Table 3) indicates that among the cattle owners who were interviewed, 99.1% (107/108) grazed their cattle in communal pastures while 98.1% (106/108) of respondents reported animals sharing water sources among different herd groups of livestock and wildlife. Furthermore, 88.9% (96/108) of the households reported contact/interaction of their livestock with wildlife (Table 3), particularly at water sources. In addition to that, 84.3% (91/108) of the respondents moved animals close to protected areas within the ecosystem (Serengeti National Park, Ikorongo/Grumeti Game Reserve, Loliondo Game Controlled Area and Ngorongoro Conservation Area) in search of pastures especially during the dry season. Moreover, 98.1% (106/108) of respondents reported that the main source of water for their livestock is shared or communal sources. The awareness of respondents to bovine tuberculosis was very low and 64.8% of the respondents had never heard about bovine tuberculosis. Likewise, respondents had poor knowledge concerning transmission pathways of bovine tuberculosis, where 39.5% only had an idea on how the disease is spread. On the other hand, 80.6% were not aware whether bTB is present in wildlife or not. Of the respondents, 64.8% (70/108) reported the presence of very thin and emaciated animals in their herds. However, only 13% of the respondents reported condemnations of tissues suggestive of bTB infection when it happens that pastoralist slaughter their animals at their vicinity under assistance of veterinary service providers. In general, 97.2% of the respondents indicated that they receive at least some veterinary services to their livestock, including dipping, treatment of diseases and regular administration of antihelminthics. Assessment of herd size in relation to number of positive animals by descriptive statistics indicated that herd sizes were not normally distributed. Therefore a non parametric analysis (Kruskal-Wallis Test) was used to find out whether we could predict how many herds could be positive out of 32. However, the test showed insignificant results (χ^2^ = 3.12, *P* = 0.08) therefore, we could not predict how many herds we might expect to be positive of the 32 considered.

**Table 3:** Results of questionnaire on risk factors and awareness of cattle owners on bovine tuberculosis

**Category**	**Variable**	**Level**	**Responses**	
			**n**	**%**
Practices	Types of grazing system	Communal pasture	107	99.1
		Communal/own pasture	0	0
		Own field/paddocks	1	0.9
	Receiving veterinary service	No	3	2.8
		Yes	105	97.2
	Contact of livestock with wild animals at water sources	No	12	11.1
		Yes	96	88.9
	Moving animals close to protected areas searching for grazing land	No	17	15.7
		Yes	91	84.3
	Source of water for livestock	Shared/communal	106	98.1
		Own/communal watering points	2	1.9
Knowledge of bTB	Heard about bovine tuberculosis	No	70	64.8
		Yes	38	35.2
	If yes, knowledge on transmission	No	15	39.5
		Yes	23	60.5
	Awareness of bTB in wildlife	No	87	80.6
		Yes	21	19.4
	Presence of any coughing animal in the herd	No	60	55.6
		Yes	48	44.4
	Presence of very thin and emaciated animals in the herd	No	38	35.2
		Yes	70	64.8
	Presence of both emaciated and coughing animals in the herd	No	54	50.0
		Yes	54	50.0
	Condemnation of a lung with nodular bTB like lesions	No	94	87.0
		Yes	14	13.0

Owners (n = 108) on bovine tuberculosis in cattle and wildlife.

## Discussion

This study has shown an overall bTB apparent prevalence of 2.4% in cattle around the Serengeti ecosystem. Generally the prevalence in this study is consistent with studies conducted in other regions of Tanzania [5,11,13-15] and like for other areas previously studied, bTB is endemic. Results from this study show low prevalence of bTB as compared to a previous study by Kazwala et al. [17] in Southern Highlands of Tanzania. This discrepancy could be attributed to difference in sample sizes and study design. The herd prevalence of 50% (16 out of 32) might reflect that bTB infection varies considerably between herds and is widespread in pastoral and agro-pastoral communities in the Serengeti ecosystem and surrounding areas.

The study has also shown that most of the cattle owners interviewed in villages had poor knowledge on bTB. Moreover, the majority of the respondents had never heard of bovine tuberculosis and were not aware of bTB presence in wildlife. The infection of pastoral and agro-pastoral cattle with bTB, poor community knowledge on the mode of transmission for bTB among people poses serious risks of infection with zoonotic diseases including bTB to people in the Serengeti ecosystem. Therefore, there is a great need for awareness creation and community involvement in planning and implementation of disease control programmes especially zoonotic diseases in the Serengeti ecosystem.

The prevalence of bTB among the three districts varied considerably with significant differences between the three districts (Table 2). Although our point estimates of prevalence in the three districts varied five-fold, the sample size was not large enough to be sure that these are significant differences. The relatively high proportion of bTB infection in Ngorongoro district compared to other two districts might be due to husbandry practices of semi-nomadic system where the Maasai pastoralists move their cattle during the dry season searching for water and pasture [40], and multiple land use system in Ngorongoro where human, livestock and wildlife live in the same area [31]. These practices predispose cattle to bTB infection due to higher chances of coming in contact with infected animals. Nevertheless, it is proposed that bTB occurs in hotspots [41]. The difference in bTB prevalence among hotspots are influenced by ecological factors, cultural factors, livestock trade patterns, host populations, timing of contact and reproductive rate of pathogens [41-43].

Previous studies conducted in wildlife in Serengeti have confirmed the presence of *M. bovis* in buffalo, wildebeest and lion [5]. The buffalo is considered as the natural host of *M. bovis* and its social behaviour of living in large herds provides favourable conditions for aerosol transmission of *M. bovis* to other members of the same herd [44]. The migration of wild animals traversing the Serengeti ecosystem heading northwards to Maasai Mara in Kenya could contribute to the spread of bTB to the nearby surroundings’ villages. For example, 88.9% of the cattle owners interviewed in this study reported contact of cattle with wildlife at water sources. In this study, there was a significant association between age and intradermal skin positivity with more positive animals in medium aged groups (2–4 years). These are weaners which are likely to get exposed to infection with time and remain sensitised to bovine tuberculin for the rest of their life [40]. While young animals are unlikely to get infected with *M. bovis*, it is said that age alone is not enough to account for susceptibility to infection but continued opportunities to exposure to the bacterium. Previously published studies conducted in the Southern Highlands and northern regions of Tanzania [5,17] indicated a trend-wise increase in positive reactivity as age increases which was not the case for our study. Existing belief is that, in endemic situation, the duration of exposure to bTB infection increases with age [5,17,24]. Similarly, the size of the households correlated to intradermal skin positivity with more reactors in medium sized households. Despite the lack of a clear explanation for this association, other risk factors can play a role as pointed out in a stratified classification of worldwide bovine tuberculosis risk factors in cattle [2]. For example animals regardless of size of the household herdsmen could move their animals to longer distances in search of pasture and water sources. In so doing chances of meeting infected herds including wildlife may occur thus exposing to infection.

Findings from this study have indicated no significant association between sex and intrademal skin positivity (Table 1). The finding concurs with previous studies conducted in northern regions of Tanzania and elsewhere, which reported similar findings for bTB positivity between male and female cattle [3,5]. Contrary to our findings, other studies have reported an association between sex and intradermal skin positivity [17,24,45]. For example Inangolet *et al*. [45] and Cadmus *et al*. [24] reported female cattle being at greater risk of testing positive than males contrary to findings by Kazwala *et al*. [17], where male cattle were more affected than female cattle as male cattle particularly castrates are kept longer and hence more chances of contracting a disease than female cattle. In controlled studies no difference in susceptibility to bTB in relation to gender/sex has been found (Anita Michel, personal communication). It is probably more the likelihood of production type (e.g. dairy cows) and exposure that can cause a difference, e.g. breeding bulls which are shared between farmers.

Our results have shown that about 64.8% of respondents reported presence of very thin and emaciated animals in their herds. However, through physical observation, most of thin and emaciated animals in those herds tested negative for bTB as compared to positive reactors whose body condition was good. This supports previous findings by Amen *et al*. [22] and Munyeme *et al*. [46] in which most of positive reactors cattle had good body condition as compared to negative reactors. With exception of animals in advanced stages of bTB when emaciation and laboured breathing are more prominent clinical signs, body condition has a direct association with individual health condition and it is probably not a reliable determinant for bTB infection. During this study, it was interesting to note that some very healthy animals in Bunda district, although had less bTB overall as compared to other districts, tested positive to tuberculin skin test. The good body condition score (even for infected cattle) in Bunda district might be due to quality and plentiful pastures and water for cattle, making animals look healthier than in Serengeti and Ngorongoro districts. Poor body condition to animal herds in Ngorongoro districts could possibly be attributed by drought condition in Maasai steep lands and long trekking distance where animals move long distances every day searching for pastures and water sources. Experience in human TB, the negative tuberculin skin testing (TST) (the equivalent of bTB infection) is often seen in people with overwhelming infection or disease, or with measles, HIV or malnutrition [47,48]. In our study site bTB is not routinely tested in cattle at slaughter houses for association of TB lesions with emaciation. Available reports do not indicate whether the same is true of cows and whether some of these emaciated animals have false negative reactions.

The study has shown a high prevalence of atypical mycobacteria in cattle, which signifies environmental contamination. Cattle could have acquired the infection through contaminated environment during grazing or at water sources. The high prevalence of atypical mycobacteria in this study concurs with previous findings in Tanzania whereby Shirima *et al*. [13] and Durnez *et al*. [15], reported a prevalence of 6% and 10.1% of atypical mycobacterioses respectively. In their study Mdegella *et al*. [14] and Durnez *et al*. [15] found a prevalence of 14% and 19% of atypical mycobacteria in milk samples that could expose milk consumers at great risk of contracting milkborne zoonotic infections. Consumption of undercooked meat and unpasturalized milk is a common practice in most pastoral communities in Tanzania [17]. The prevalence of 10.6% of atypical mycobacteria obtained in this study may alert for a possibility of immune compromised individuals such as HIV/AIDS patients and puts them into a risk of being infected with opportunistic infections. This is particularly critical if at all the atypical mycobacteria such as *M. avium* and *M. fortuitum* are shed in meat or milk from cattle as it happens in case of *M. bovis*. Atypical mycobacteria which are commonly environmental have been reported in cattle farms [49] with wide distribution in nature (soil, water, animals and humans).

Available reports revealed atypical mycobacteria to include *M. gordonae, M. smegmatis, M. fortuitum, M. phlei, M. flavescenes* and *M. avium intracellular* to be shed in cattle milk samples in Morogoro region and Kibaha, Tanzania [14]. *M. avium* is said to be clearly a human pathogen in infants and also in HIV-infected adults; but the others are arguably less virulent, with *M. fortuitum* very rarely causing disease in individuals with genetic immune system problems. Others are regarded to be commensals. However, the non-specific reactions are not always caused by atypical mycobacteria. Sometimes, other closely related bacteria species (Nocardia, Corynebacterium, Trueperella etc.), as well as some other factors can cause the unspecific reactions (Anonymus, 2013).

The study has shown that older cattle (>4 years) had a high response to PPD and doubtful reaction as compared to young cattle, with more doubtful reaction in females as compared to males (Figure 4). It is believed that doubtful reaction in older cattle might be due to immune suppression owing to old age [45]. Furthermore, it is suggested that, stressful conditions such as drought conditions, return of animals from transhumance, long trekking, clinical ill-health due to trypanosomiasis, tick borne diseases, ectoparasites, heavy burden of endoparasites and other environmental stressors could contribute to higher rates of doubtful reactions [50]. However, in this study the doubtful reactors were not re-tested after 60 days as per International Office of Epizootics (OIE) guidelines due to time limitation and field logistics.

## Conclusions

In conclusion, findings from this study add useful epidemiological data/information regarding bTB infection at the livestock-wildlife interface in Tanzania. It was revealed that agro-pastoral and pastoral communities in the study area have low knowledge on bTB and its presence in other animals including wildlife. Moreover, majority of the community engage in husbandry practises such as free movement of cattle, sharing of water sources and pasture which increase chances of diseases transmission to livestock. This information is useful and could be used by respective authorities in designing appropriate control measures against bTB. Therefore, we propose that education programs should be implemented in pastoral communities to raise awareness on preventive measures against bTB and other infectious diseases. The education programs at livestock-wildlife interface areas should primarily focus on minimizing contact between cattle and wildlife. However, in order to be more certain whether strains are circulating between cattle and wild animals, we need to cultivate mycobacteria and use genetic strain typing methods to characterise them accurately.

## Competing interests

The authors declare that they have no competing interest related to this article.

## Authors’ contributions

BZK designed study, conducted field work, analyzed the data, drafted the manuscript. EVM participated in designing the study, supervised the field work and contributed in drafting and initial review of the manuscript. EDK assisted in initial design of the study and reviewed the manuscript. JDK contributed to conception and commented the manuscript. SK assisted in conceptualization and critically reviewed the manuscript. GSK assisted in critical review of the manuscript and commented the manuscript. PGF had an important contribution and commented the manuscript. ALM contributed to conception and critically commented the manuscript. RRK contributed in outset and review of the manuscript. PVH made conceptual contribution commented the manuscript and critically revised it. MIM coordinated the field work and helped in drafting the manuscript. All authors have read and approved the final manuscript.
